# Impact of Photon Counting Detector CT Derived Virtual Monoenergetic Images on the Diagnosis of Pulmonary Embolism

**DOI:** 10.3390/diagnostics12112715

**Published:** 2022-11-07

**Authors:** Tetyana Yalynska, Malgorzata Polacin, Thomas Frauenfelder, Katharina Martini

**Affiliations:** Diagnostic and Interventional Radiology, University Hospital Zurich, University of Zurich, 8091 Zurich, Switzerland

**Keywords:** computed tomography, photon-counting, pulmonary embolism, image processing, quality evaluation, virtual monoenergetic image, thorax

## Abstract

**Purpose:** To assess the impact of virtual-monoenergetic-image (VMI) energies on the diagnosis of pulmonary embolism (PE) in photon-counting-detector computed-tomography (PCD-CT). **Methods:** Eighty patients (median age 60.4 years) with suspected PE were retrospectively included. Scans were performed on PCD-CT in the multi-energy mode at 120 kV. VMIs from 40–70 keV in 10 keV intervals were reconstructed. CT-attenuation was measured in the pulmonary trunk and the main branches of the pulmonary artery. Signal-to-noise (SNR) ratio was calculated. Two radiologists evaluated subjective-image-quality (noise, vessel-attenuation and sharpness; five-point-Likert-scale, non-diagnostic–excellent), the presence of hardening artefacts and presence/visibility of PE. **Results:** Signal was highest at the lowest evaluated VMI (40 keV; 1053.50 HU); image noise was lowest at the highest VMI (70 keV; 15.60 HU). Highest SNR was achieved at the lowest VMI (*p* < 0.05). Inter-reader-agreement for subjective analysis was fair to excellent (k = 0.373–1.000; *p* < 0.001). Scores for vessel-attenuation and sharpness were highest at 40 keV (both:5, range 4/3–5; k = 1.000); scores for image-noise were highest at 70 keV (4, range 3–5). The highest number of hardening artifacts were reported at 40 keV (*n* = 22; 28%). PE-visualization was rated best at 50 keV (4.7; range 4–5) and decreased with increasing VMI-energy (r = −0.558; *p* < 0.001). **Conclusions:** While SNR was best at 40 keV, subjective PE visibility was rated highest at 50 keV, potentially owing to the lower image noise and hardening artefacts.

## 1. Introduction

Pulmonary embolism (PE) is defined as an occlusion of the pulmonary arteries by thromboembolic material [1,2,3,4,5,6]. It is a potentially life-threatening condition which frequently results in death [1,2,3,4,5,6]. PE contributes to one of the most common in-hospital complications, causing widespread morbidity and mortality [1,7]. Accurate and timely diagnosis of PE is crucial for appropriate treatment and ultimately, for improvement of the patients’ outcome [7]. Multi-detector computed tomography (CT) has demonstrated high diagnostic accuracy in the diagnosis of PE, hence becoming the modality of choice for patients with suspected PE [1,2,3,4,5,6,7,8,9,10]. The quality of the images during routine clinical use of CT angiography (CTPA) is dependent on several factors including bolus timing, breathing-related effects, iodine delivery rate (iodine contrast agent concentration and injection flow-rate) and low cardiac output [1,8,11]. Such technical and patient-dependent factors can lead to suboptimal image quality and undermine the PE diagnosis [4,11].

A new and rapidly developing generation of CT scanners significantly improves and expands the diagnostic possibilities of the method [9,10,12]. Photon-counting detector CT (PCD-CT) can provide energy-resolved CT data at very high spatial resolution, with significantly decreased electronic noise, as well as radiation dose reduction [12,13,14]. This new technology can help to overcome the major limitations of conventional CT detectors, such as limited spatial resolution and electronic noise [15,16,17,18]. Together, the multi-energy data acquisition and the reconstruction of virtual monoenergetic images (VMI) at varying energies allow for the adjustment of the level of energy to provide the optimal combination of signal and image noise [16,19,20,21] for the diagnosis in question. Further, VMI allows for the creation of virtual low keV datasets, with which the attenuation of the iodine contrast and signal-to-noise ratio can be optimized for the evaluation of vascular structures. Using VMI has already been beneficial for improving image quality and visualization of vascular structures in PCD-CT [4,5,21,22,23].

There are studies which evaluate the impact of different VMI for the diagnosis of PE on dual-energy CT (DECT) [4,5]. However, there is a lack of literature addressing the use of VMI for the evaluation of PE in PCD-CT.

This study aimed to fill this gap by investigating the impact of VMI at different VMI energies in the diagnosis of PE.

## 2. Materials and Methods

### 2.1. Patient Cohort

This retrospective study was approved by the local ethics committee (approval number 2019-01676). Written informed consent was obtained from all patients. Consecutive patients who were referred to our institution for ruling out PE and obtained a dedicated contrast enhanced thoracic CT angiography between March 2021 and June 2022 on a first-generation dual-source PCD-CT were retrospectively included. Diagnosis of PE was indicated on the direct visualization of hypoattenuating filling defects in the enhancing lumen of the pulmonary artery [2]. Of the initially included patients, all with a diagnosis of PE were added to the final study cohort. Additionally, we added 45 consecutive patients without PE. The residual patients without PE were dismissed.

### 2.2. Image Acquisition

PCD-CT scans were performed on a first-generation dual-source CT scanner with “Quantum technology” (NAEOTOM Alpha, Siemens). This scanner is equipped with a photon-counting detector (PCD) (QuantaMax detector, Siemens). All scans were performed in the multi- energy (QuantumPlus) mode at 120 kVp at an image Quality (IQ) level of 60 after application of an intravenous (IV) iodinated contrast agent.

A double-syringe power injector (CT Exprés, Bracco, Lausanne, Switzerland) applied IV contrast via an antecubital vein. 80 mL of IV contrast (iopromide, Ultravist, 300 mg J/mL, Bayer HealthCare, Leverkusen, Germany) was followed by 50 mL saline bolus, both at a flow rate of 4 mL/s. Bolus tracking was performed with a threshold of 100 HU (at 100 kVp) in the main pulmonary artery, with a trigger delay of 10 s.

### 2.3. Post Processing

All images were reconstructed in the axial plane with a soft tissue convolution kernel (Br36) at a slice thickness of 2 mm and an increment of 1.6 mm at different VMI energies, ranging from 40 to 70 keV with a stepwise increment of 10 keV and multi-energetic images containing all energy spectra (SPP).

### 2.4. Objective Image Quality Evaluation

One reader blinded for the reconstructed VMI energy measured the CT attenuation by placing circular regions of interest (ROIs) in the pulmonary trunk and the main branches of the pulmonary artery. For each anatomical structure, an area with the largest possible ROI diameter was chosen. ROIs were only placed in positions were no image artifacts or filling defects of the pulmonary arteries were present. Image noise was defined as the standard deviation of air attenuation in the trachea just above the bifurcation. Signal- to-noise ratio (*SNR*) was calculated as follows:

(1) $$SNR=\frac{HU_{Pulmonary\;artery}}{Noise}$$

### 2.5. Subjective Image Quality Evaluation

Subjective image quality evaluation was performed by two independent readers (TY, with 16 years, and MP, with 9 years of experience in thoracic imaging) blinded for the reconstructed VMI energy. Readers had to score subjective image noise, vessel attenuation and vessel sharpness on a 5-point Likert scale (1 = non-diagnostic, 2 = poor, 3 = moderate, 4 = good, 5 = excellent). Readers further had to state if hardening artefacts due to contrast agent were present (yes/no).

### 2.6. Detection of Pulmonary Embolism

The same two blinded readers had to evaluate if pulmonary embolism was present (yes/no) and if present, to score the visibility of PE (1 = non-diagnostic, 2 = poor, 3 = moderate, 4 = good, 5 = excellent). Readers were allowed to change window levels.

### 2.7. Statistical Analysis

All data distribution was initially checked visually (histograms, boxplots, quantile-quantile plots) and then quantitatively (Shapiro-Wilk tests) for normal distribution. Quantitative variables were expressed as median and ranges with a 95% CI. Cohen’s κ was used to assess inter-reader agreement. The following scale was used for the indication of the level of agreement by k-results: slight agreement, 0.01–0.20; fair agreement, 0.21–0.40; moderate agreement, 0.41–0.60; good agreement, 0.61–0.80; excellent agreement, 0.81–0.99) [24]. A 2-sided pairwise comparison was used to compare median variables. Bonferroni correction was used to correct for multiple tests. A two-sided *p*–value below 0.05 was considered to indicate statistical significance. Statistical analyses were conducted using commercially available software (IBM SPSS Statistics, release 21.0; SPSS, Chicago, IL, USA).

## 3. Results

### 3.1. Patient Population

From March 2021 to June 2022, in total three hundred fourteen patients underwent imaging on a PCD-CT with the suspicion of PE. In our study we included all patients with the diagnosis of PE (*n* = 35, 11.1%) (Table 1) and 45 consecutive patients with unremarkable pulmonary arteries in order to obtain homogeneous groups. The final study cohort constituted eighty patients (median age: 60.4 years; range: 19.5–90 years; 28 women). The two groups did not show statistically significant differences based on gender and age (*p* > 0.05).

### 3.2. Objective Image Quality Evaluation

**Figure 1:**
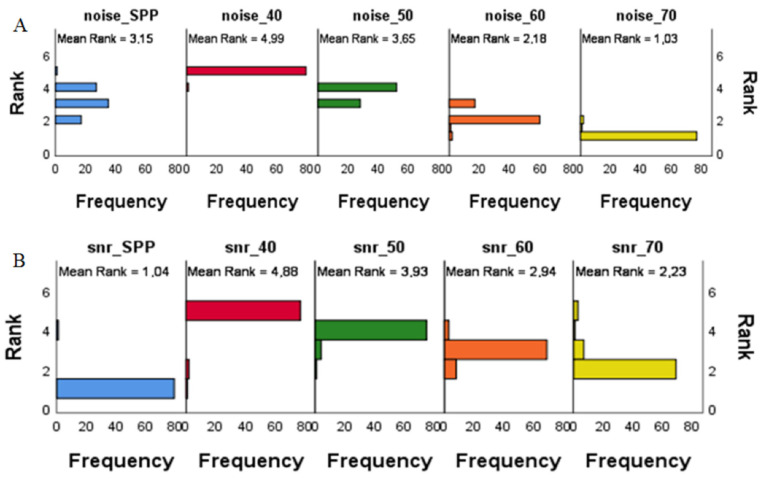
Bar plots of the frequency of variance by ranks for image noise (**A**) and Signal-to-Noise ratio (SNR) (**B**) at different virtual monoenergetic image (VMI) energies. The lowest ranks for image noise were achieved at the highest evaluated VMI energy (70 keV); (**A**) the highest ranks for SNR were achieved at the lowest evaluated VMI energy (40 keV); (**B**) SPP—multi-energy image with all energy spectra.

Overall, pulmonary artery attenuation, image noise and SNR did not have a statistically significant difference for patients with and without PE (*p* > 0.05). The lowest values for pulmonary artery attenuation and image noise were achieved at the highest evaluated VMI energy (70 keV), while the highest values for SNR were achieved at the lowest evaluated VMI energy (40 keV). Values for image noise and SNR can be found in Figure 1 and Table 2.

The pairwise comparison of SNR at different keV indicated a statistically significant difference between all combinations of VMI energies (*p* = 0.044–0.000) (Table 3); the closest values were SNR 60/70 (*p* = 0.044). The pairwise comparison for noise values at different keV had a statistically significant difference between 40/50 keV, 50/60 keV, 60/50 keV, 60/70 keV (*p* < 0.001) (Table 3).

Signal and noise decreased with higher VMI energies (*p* < 0.001), while SNR had a negative correlation with increasing VMI energies (r = −0.635; *p* < 0.001).

### 3.3. Subjective Image Quality Evaluation

**Figure 2:**
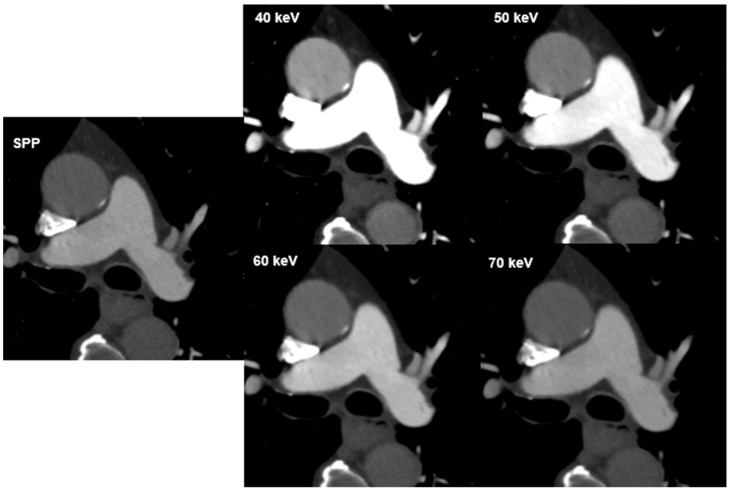
CT images of a 72-year-old male patient at different virtual monoenergetic image (VMI) energies and the multi-energy reconstruction, including all energy spectra (SPP). The visual difference in image quality. Attenuation in the pulmonary artery and aorta depends on the image energy and decreases with increasing VMI energy.

Overall, inter-reader agreement for subjective image quality was moderate to excellent (Table 4). Excellent agreement was reached in the evaluation of vessel attenuation on VMIs 40–70 keV (k = 0.857–1.000) with total consensus for VMI 40 keV and 50 keV (k = 1.000) and good agreement was reached in the evaluation of vessel sharpness (k = 0.717–0.830). The agreement in image noise evaluation on VMI 40 keV was moderate (k = 0.584), good agreement was achieved for VMIs 50–70 keV (k = 0.644–0.682). Vessel attenuation and vessel sharpness were rated best at 40 keV (both mean 5; range 3–5), while image noise was rated best at 70 keV (mean, 4.6; range 3–5). Subjective scores in the assessment of image noise had positive correlation with VMI energies (r = 0.231, *p* < 0.001), while there was a negative correlation between VMI energies and vessel attenuation and sharpness (r = −0.557 and r = −0.280; *p* < 0.001). Hardening artefacts were mainly reported at 40 keV (*n* = 22; 27.5, followed by 50 keV with *n* = 10 (12.5%) and 60 keV with *n* = 1 (1.3%). No hardening artefacts were reported at 70 keV. The visual difference in image quality with regard to image noise and signal is illustrated in Figure 2.

### 3.4. Detection of Pulmonary Embolism

**Figure 3:**
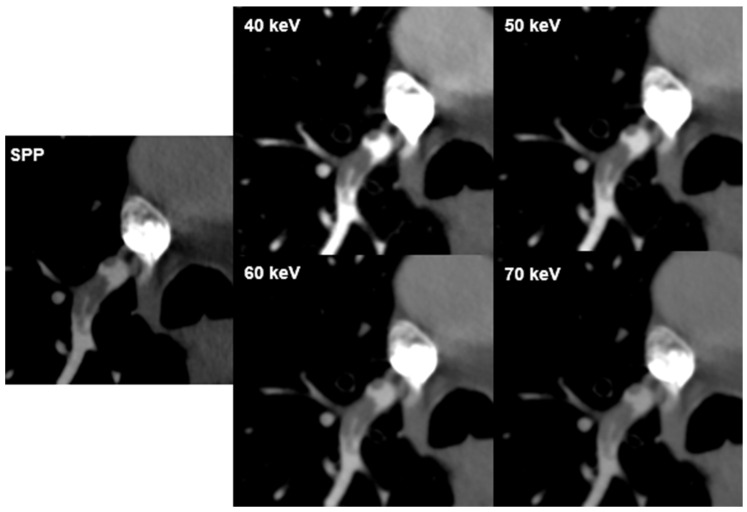
PE visualization. CT images of a 73-year-old male patient with pulmonary embolism (PE) in a branch of the right pulmonary artery in the lower lobe at different virtual monoenergetic image (VMI) energies and the multi-energy reconstruction, including all energy spectra (SPP). Attenuation in the pulmonary artery depends on the image energy. PE is best seen at 40 keV and 50 keV.

Inter-reader agreement for PE visualization was fair to good (k = 0.373–0.612, *p* < 0.001) with good concurrence for VMI 50 keV (k = 0.612). Diagnostic confidence for the visualization of PE was rated lowest at 70 keV for both readers (mean 3.7; range 2–5 and 3.5; range 2–4; respectively). There was a negative correlation between PE visualization and VMI energies for both readers (r = −0.538 and r = −0.578, *p* < 0.001; respectively) (Figure 3).

## 4. Discussion

Current publications spotlight many benefits of the PCD-CT in comparison with previous generations of CT; they provide a higher contrast-to-noise ratio, lower radiation dose and the possibility to reconstruct VMI energies [12,13,15,16,17,19,25,26,27,28]. Our study assessed the impact of different VMI energies in the diagnosis of PE with PCD-CT. While the use of low-energy VMI energies is improving, vessel attenuation in the aorta has already been widely evaluated [25]; however, the impact of VMI in PCD-CT on the evaluation of the pulmonary artery and PE has not yet been evaluated. The value of VMI in DECT datasets for improving the quality of CT pulmonary angiography (CTPA) examinations has been described in the literature over the recent six to eight years [1,3,4,5,8,9,21,22,23,29,30]. Albrecht et al. summarized CTPA techniques current at the time of publication, advanced postprocessing application to enhance image quality, and discussed the appropriate use of CT for acute PE detection [9]. Hong et al. in a review article reported that DECT is useful for detecting PE, although DECT is not regarded as routine diagnostic procedure in the evaluation of PE according to current guidelines [2].

In our study, the lowest values for pulmonary artery attenuation and image noise were achieved at the highest evaluated VMI energy (70 keV). The highest values for pulmonary artery attenuation, image noise and SNR were seen at the lowest evaluated VMI energy (40 keV). Our data are consistent with the predicted outcome for the imaging technology of VMI and similar to data from Bae et al. [30].

VMI yields results similar to single energy X-ray beams; the images are attained through linear combination of basis pair images; for example, low and high energy images can be combined at various proportions [10]. There is a relationship between the VMI energy and the amount of signal and image noise produced. It is known that VMI at 70 keV is considered to provide equivalent CT attenuation values of a conventional 120 kVp multi-energetic image, with similar attenuation values [10]. Therefore, as the VMI energy increases (>70 keV), the signal of contrast decreases, leading to lower image signal and noise. The opposite is true when the VMI energy is decreased (<70 keV). Our lowest evaluated VMI energy of 40 keV came closest to the K-edge value of iodine and resulted in the highest CT attenuation [10,20,22].

While in this study, vessel attenuation was rated best at the lowest-evaluated VMI energy (40 keV), PE visualization was rated best at VMI 50 keV. This might be owing to the higher image noise and number of hardening artefacts at 40 keV compared to 50 keV. Additionally, hardening artefacts from surrounding contrast agents might influence PE visibility. Rajiah et al. published a review of the technique and applications of multi-energy CT in an evaluation of pulmonary vasculature and concluded that VMI images at low energies are utilized to enhance vascular contrast, while VMI images at high energies allow for the reduction of image noise [10]. Our results are also in line with studies that evaluated different CTPA protocols in DECT, reporting that the optimal VMI energy in cases of incidentally detected PE are between 40 keV and 55 keV [31]. Dane et al. determined the optimal image energy for pulmonary artery evaluation on DECT and found that mean SNR and subjective image quality evaluation were highest at 40–50 keV [8]. Another study demonstrated that VMI 40 keV showed the highest detectability of PE and vessel attenuation in DECT [32]. In line with our study, Albrecht et al. found that the most suitable image energy for the evaluation of pulmonary arteries and diagnosis of PE in DECT is 50 keV due to contrast optimization with respect to CT attenuation and noise [23].

Different studies have found substantially increased image noise for VMI at 40 and 50 keV [33,34,35]. The authors noted that VMI 70 keV were subjectively preferred [33,34,35]. Similarly, in our study the subjective assessment of image noise was best at VMI energy 70 keV.

Another important point, if PE detection is clinical, is in the assessment of probability scores such as the Geneva or Wells score. Probability scores give information on the likelihood of a PE in a certain patient, and therefore the need if PE evaluation with CT is needed or not [36].

Our study has the following limitations: First, the study was limited to 80 patients (35 patients with PE) and the objective assessment of only three objective values (image noise, pulmonary artery attenuation and SNR) in the soft tissue kernel. Second, we did not evaluate the diagnostic accuracy of PE detection, but both readers had absolute agreement on the presence on PE. Third, subjective image quality analysis was only performed by two readers. Fourth, in PCD-CT the user has to choose from a set of different IQ levels. Each IQ level uses different mAs settings to obtain comparable image quality throughout patients regardless of patient size/Body Mass Index etc. The different mAs value chosen individually by the scanner might also influence CT attenuation.

## 5. Conclusions

While SNR was best at 40 keV, subjective PE visibility was rated highest at 50 keV potentially owing to the lower image noise and lower number of hardening artifacts.

## Figures and Tables

**Table 1:** Distribution of pulmonary embolism in study cohort.

PE	*n* (%)
Central	2 (5.7)
Lobar	18 (51.4)
Segmental	19 (54.3)
Subsegmental	23 (65.7)
Unilateral	18 (51.4)
Bilateral	17 (48.6)

Pulmonary embolism (PE), number of cases (*n*). In patients where PE distribution involves more locations (from center to periphery), each location was counted separately.

**Table 2:** Objective image quality indices at different virtual monoenergetic image (VMI) energies (keV).

Imaging Parameter	SPP	40 keV	50 keV	60 keV	70 keV
Image noise median CI95% [range]	20.85 [19.5–21.80]	27.25 [25.80–29.85]	21.70 [20.60–22.70]	17.65 [17.25–19.00]	15.60 [14.30–16.45]
Signal median CI95% [range]	358.83 [340.67–393.66]	1053.50 [976.01–1146.33]	703.16 [655.36–748.00]	488.33 [459.67–536.33]	358.83 [341.00–394.65]
SNR median CI95% [range]	17.78 [14.29–20.13]	37.56 [32.48–40.48]	33.18 [27.83–35.08]	28.06 [23.60–30.78]	23.61 [19.84–27.04]

Values are given as Median CI95% [low-high range]; Signal-to-noise ratio (SNR); multienergy reconstruction including all energy spectra (SPP).

**Table 3:** Pairwise Comparisons of median SNR and image noise at different VMI energies.

SNR	*p* ^a^	Image Noise	*p* ^a^
40/50	0.001	40/50	0.000
50/60	0.001	50/60	0.000
60/70	0.044	60/70	0.000
40/70	0.000	40/70	0.000
50/70	0.000	50/70	0.000
40/60	0.000	40/60	0.000

The significance level is 0.05. Signal to noise ratio (SNR). ^a^ Bonferroni correction was performed for multiple tests.

**Table 4:** Subjective analysis result. Qualitative image assessment of image noise, vessel attenuation, vessel sharpness and PE visualization at different VMI energies with a five-point Likert scale.

Imaging Parameter	VMI Energies, keV	R1 (Mean, Range)	R2 (Mean, Range)	Cohen’s Kappa (k)	*p*	*n*
Image noise	40	4.1 [3–5]	4.1 [3–5]	0.584	0.000	80
	50	4.2 [3–5]	4.3 [3–5]	0.681	0.000	80
	60	4.4 [3–5]	4.4 [3–5]	0.644	0.000	80
	70	4.6 [3–5]	4.6 [3–5]	0.682	0.000	80
	SPP	4.8 [3–5]	4.6 [3–5]	0.644	0.000	80
Vessel attenuation	40	5.0 [4–5]	5.0 [4–5]	1.000	0.000	80
	50	4.9 [4–5]	4.9 [4–5]	1.000	0.000	80
	60	4.5 [3–5]	4.5 [3–5]	0.977	0.000	80
	70	4.2 [3–5]	4.1 [3–5]	0.899	0.000	80
	SPP	4.0 [3–5]	4.1 [3–5]	0.593	0.000	80
Vessel sharpness	40	4.7 [3–5]	4.8 [4–5]	0717	0.000	80
	50	4.5 [3–5]	4.7 [4–5]	0.794	0.000	80
	60	4.3 [4–5]	4.7 [4–5]	0.830	0.000	80
	70	4.2 [3–5]	4.6 [3–5]	0.783	0.000	80
	SPP	4.2 [3–5]	4.3 [3–5]	0.783	0.000	80
PE visualization	40	4.5 [3–5]	4.4 [3–5]	0.515	0.001	35
	50	4.7 [4–5]	4.7 [4–5]	0.612	0.000	35
	60	4.1 [3–5]	3.8 [3–5]	0.392	0.001	35
	70	3.7 [2–5]	3.5 [2–4]	0.373	0.001	35
	SPP	3.8 [2–5]	3.4 [2–4]	0.373	0.000	80

The data is presented as mean (interquartile range) for Reader 1 (R1) and Reader 2 (R2), respectively. (*p*—the significance level < 0.05.) VMI—virtual monoenergetic images; PE—pulmonary embolism, multi-energy reconstruction including all energy spectra (SPP).

## Data Availability

Data are available upon request.
